# Water Dynamics in Highly Concentrated Salt Solutions: A Multi‐Nuclear NMR Approach

**DOI:** 10.1002/open.202200080

**Published:** 2022-05-31

**Authors:** Nasrollah Rezaei‐Ghaleh

**Affiliations:** ^1^ Institute of Physical Biology Heinrich Heine University Düsseldorf Universitätsstrasse 1 40225 Düsseldorf Germany; ^2^ Institute of Biological Information Processing IBI-7: Structural Biochemistry Forschungszentrum Jülich 52428 Jülich Germany

**Keywords:** diffusion, dynamics, quadrupolar NMR, salt, water

## Abstract

Living cells often contain compartments with high concentration of charged biomolecules. A key question pertinent to the function of biomolecules is how water dynamics are affected by interaction with charged molecules. Here, we study the dynamical behavior of water in an extreme condition, that is, in saturated salt solutions, where nearly all water molecules are located within the first hydration layer of ions. To facilitate disentangling the effects of cations and anions, our study is focused on alkali chloride solutions. Following a multi‐nuclear NMR approach enabling direct monitoring of protons and the quadrupolar nuclei ^7^Li, ^17^O, ^23^Na, ^35^Cl, ^87^Rb and ^133^Cs, we investigate how the translational and rotational mobility of water molecules, chloride anion and corresponding cations are affected within the constrained environment of saturated solutions. Our results indicate that water molecules preserve a large level of mobility within saturated alkali chloride solutions that is significantly independent of adjacent ions.

## Graphical abstract

**A multi‐nuclear NMR approach** including quadrupolar nuclei is used to monitor water and ions mobility in a series of saturated salt solutions. It is shown that water can preserve a high degree of mobility inside such solutions. The results shed light on water dynamics in highly crowded environments such as phase‐separated cellular compartments enriched with charged biomolecules.

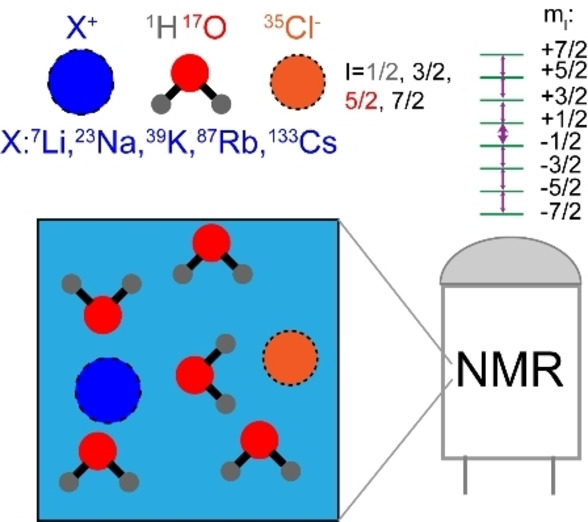

## Introduction

Liquid water is a highly abundant material on earth and covers around 70 % of earth surface.^[1]^ Despite some controversies, the primary source of earth water is widely believed to be carbonaceous chondrites from outer asteroid belt.^[2]^ Liquid water is the host for life as we know of it on earth and the physical conditions supporting its existence on the surface of a planet define the criteria of circumstellar habitable zone in the search for extraterrestrial life.^[3]^ Considering its ubiquitous presence and crucial importance especially in the realm of living matter, it is not difficult to understand why water was regarded as the origin and principle (“Arche, αρχη”) of nature by Thales of Miletus, arguably the founder of natural philosophy.

A key property of liquid water is that it is a highly structured liquid possessing an extensive network of hydrogen bonds. Unlike simpler liquids that typically act as hard spheres interacting through non‐directional van der Waals interactions, water molecules tend to form hydrogen bonds within a relatively open tetrahedral arrangement. Consequently, the most stable structure of liquid water at each temperature and pressure is largely dictated by the competition between tetrahedral hydrogen bonding and van der Waals interactions, respectively favoring open and compact structures.^[4]^ This structural property underlies some striking features of liquid water, for example, its cohesive and anomalous volumetric properties.[^4^, ^5^] The structural properties of liquid water have been extensively studied through X‐ray and neutron diffraction, Infrared (IR) and Raman spectroscopy, Rayleigh and Brillouin scattering and NMR spectroscopy, as well as by computational methods such as molecular dynamic (MD) simulations and quantum chemical calculations.[^4^, ^5^, ^6^] Accordingly, several measures of structure (such as stiffness, openness, order, Kirkwood dipole orientation correlation parameter *g*, heat capacity density, see Ref. [5]) have been developed to quantify the difference between water and (supposedly) less structured liquids. None of these parameters alone however seem adequate to represent the unique properties of water.^[5]^

Ions and charged molecules are ubiquitously present in aqueous intra‐ and extra‐cellular environments. Due to the large electric dipole of water molecules (≈2.95 D in liquid water at 300 K) and their considerable polarizability (≈1.636×10^−40^ F ⋅ m^2^), the electric field around ions leads to rearrangement of nearby water molecules in one or more hydration shells, with structure and dynamics significantly different from the bulk water. Compared to cations, the electrostatic interaction of anions with hydration layer water is further strengthened by O−H⋅⋅⋅X^−^ hydrogen bonds.^[6]^ Vast amounts of experimental and computational data have been reported on water structure in hydration shells of various ions and parameters such as coordination numbers in the first (and second) hydration shells are well known for many anions and cations.[^6^, ^7^] It is however a matter of controversy whether and to which extent the effect of ions on water structure goes beyond the hydration layer(s) and extends to bulk water. While experimental evidence (primarily based on viscosity and ion mobility data) suggests that ions may increase (structure‐making or kosmotropic ions) or decrease (structure‐breaking or chaotropic ions) the hydrogen‐bonded structure of bulk water,^[5]^ more recent studies demonstrate that the effect of ions on bulk water is rather small and argue that viscosity and mobility changes could be explained by changes in the structure and effective size of hydrated ions in dependence of water exchange between hydration shells and bulk water.^[6]^

A remarkable feature of living cells is the high concentration of biomolecules inside them. Water dynamics in such crowded and frequently confined cellular environments are likely to differ from dilute solutions or pure water. Changes in water dynamics are expected to be more pronounced inside cellular organelles, where biomolecules are even further concentrated as a consequence of, for example, phase separation. Due to the high concentration of polyelectrolytes such as nucleic acids in such environments, a big fraction of water molecules are located within hydration shells. As a result, water dynamics in such environments are largely determined by the dynamical properties of water in hydration shells. This situation is reminiscent to highly concentrated electrolyte solutions, in particular saturated solutions, where the highest attainable concentration of salt ensures that nearly all water molecules are inside hydration shells and no bulk water exists. Here, we study translational and reorientational dynamics of water molecules in such environments and investigate the effect of ions on water dynamics. We focus on “saturated” solutions of a set of alkali metal chlorides, namely lithium, sodium, potassium, rubidium and cesium chloride, in order to facilitate disentangling the effects of chloride anion Cl^−^ and corresponding monovalent cations X^+^ (X: Li, Na, K, Rb, Cs) on water dynamics. The key method is NMR spectroscopy, which allows quantitative monitoring of translational diffusion and reorientational dynamics of water. In addition to the conventional ^1^H nuclei, we exploit naturally abundant quadrupolar nuclei ^17^O, ^7^Li, ^23^Na, ^35^Cl, ^87^Rb and ^133^Cs to directly probe the dynamics of water molecules and different ions (Table 1). Following a multi‐nuclear NMR approach, we will examine whether and to which extent the dynamics of water and ions become correlated within such constrained environments.

**Table 1:** Nuclear probes of water and ions mobility in saturated alkali chloride solutions studied here.

Nucleus	Spin number, I	Gyromagnetic ratio, γ ^[a]^	Quadrupole momentum, Q [barn] ^[b]^	Natural abundance [%]
^1^H	1/2	26.7519	0	99.989
^7^Li	3/2	10.3976	−0.0400(3)	92.410
^17^O	5/2	−3.6279	−0.0256(2)	0.038
^23^Na	3/2	7.0801	+0.104(1)	100.0
^35^Cl	3/2	2.624	−0.0817(8)	75.76
^87^Rb	3/2	8.7807	+0.1335(5)	27.83
^133^Cs	7/2	3.5277	−0.00355(4)	100.0

[a] Unit: 10^7^ rad ⋅ s^−1^ ⋅ T^−1^; [b] 1 barn=10^−28^ m^2^.^[8]^

## Results and Discussion

### Water‐Ion Arrangements in Saturated Salt Solutions

Saturated solutions of alkali metal chlorides, that is, LiCl, NaCl, KCl, RbCl, CsCl, were prepared by adding each salt to water at ≈5 % above their known solubility limits at room temperature, followed by intensive vortexing of the mixtures and then discarding the insoluble precipitates. Table 2 presents the (approximate) molar concentration of each salt solution and the corresponding average distance between ion centers. Considering the diameter of a water molecule *d*_w_ of ≈0.276 nm, it is evident that nearly all water molecules are located within the first hydration layer of ions and there is no space for free “interstitial” water molecules between them.^[9]^ In addition, considering the known coordination numbers of Cl^−^ and various cations in the first hydration shell,[^5^, ^7^] the ions are expected to mostly form “solvent‐shared” ion pairs. Consequently, the water molecules are simultaneously engaged in O−H⋅⋅⋅Cl^−^ hydrogen bonds and O^−δ^−X^+^ (X: Li, Na, K, Na, Rb, Cs) electrostatic interactions.[^6^, ^10^] With the charge density of monovalent cations increasing from Cs^+^ towards Li^+^ ions, the O^−δ^−X^+^ electrostatic interactions become stronger, and through an increase in the induced partial electric charges on water oxygen and hydrogen atoms (due to the finite polarizability of the water molecule), the H^+δ^⋅⋅⋅Cl^−^ hydrogen bonds are expected to be strengthened as well. Below, we investigate the influence of these water‐ions arrangements on the mobility of water molecules, chloride anions and the corresponding cations using NMR spectroscopy. It is notable that the NMR signal of water in dilute salt solutions often represents a population‐weighted average between water molecules in hydration layer(s) and bulk water, which are in rapid exchange with each other under the so‐called fast exchange regime with respect to the NMR chemical shift timescale. In saturated salt solutions studied here, however, water molecules are predominantly located within the first hydration layer of cations and chloride ions, and therefore, NMR signals of water largely represent a single ion‐bound water species.

**Table 2:** Approximate concentration and average cation–anion distances in saturated alkali chloride solutions studied here.

Salt	Conc. [g/100 mL]	Conc. [m]	Cation–anion distance [Å]	Cation radius [Å]^[a]^	Anion radius [Å]^[a]^
LiCl	84.25	19.9	2.2	0.90	1.81
NaCl	35.9	6.1	3.2	1.16	1.81
KCl	35.5	4.8	3.5	1.52	1.81
RbCl	94.25	7.8	3.0	1.66	1.81
CsCl	191	11.3	2.6	1.67	1.81

[a] The ionic radii values reported in literature show considerable variation depending on the measurement method. The reported values are crystal ionic radii from Ref. [12] and shown only for qualitative comparison.

### Cation‐Dependent Restriction in Water Mobility Detected through ^1^H and ^17^O NMR Spectroscopy

**Figure 1:**
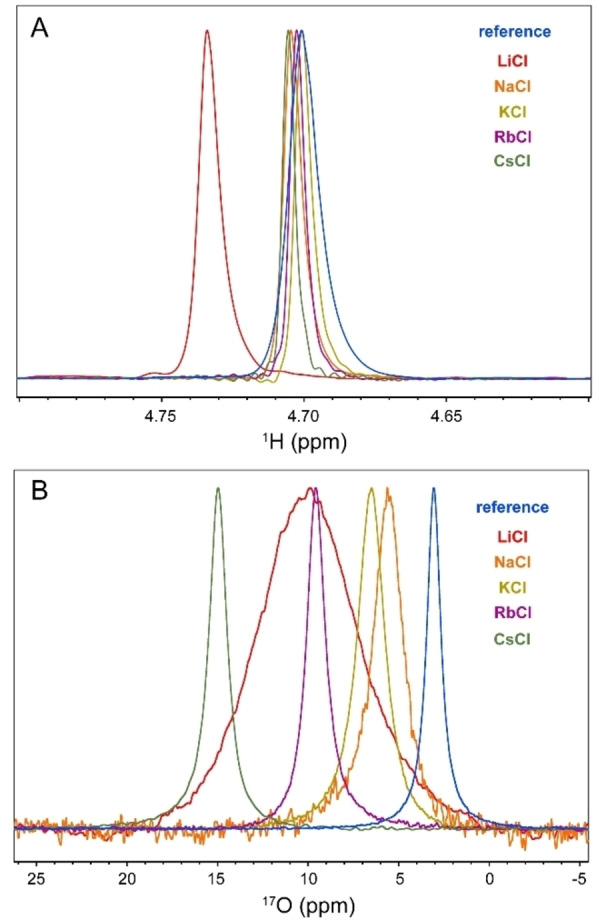
1D ^1^H (A) and ^17^O (B) spectra of reference sample (95 % H_2_O/5 % D_2_O) and saturated LiCl, NaCl, KCl, RbCl and CsCl solutions, showing cation‐dependent changes in chemical shift and signal linewidths.

Next, the 1D ^1^H and natural abundance ^17^O NMR spectra of the saturated solutions of alkali chlorides in H_2_O were measured and compared with those of reference water (containing 5 % D_2_O for frequency locking). The 1D ^1^H NMR spectra of the saturated salt solutions are shown in Figure 1A. Clearly, the different alkali chlorides had different impacts on the chemical shift and linewidths of the ^1^H signals, pointing to the role of cations on the structure and/or dynamics of surrounding water molecules.

The ^1^H signal linewidth of water is controlled by a complex set of NMR relaxation mechanisms, including intra‐ and intermolecular proton‐proton dipolar coupling, radiation damping^[11]^ and *B*_0_ inhomogeneity‐induced signal dephasing. In highly concentrated salt solutions, dipolar couplings between protons and nearby nuclei provide additional relaxation mechanisms and further complicate this picture. Unlike spin‐1/2 ^1^H nuclei, the NMR relaxation of spin‐5/2 ^17^O nuclei is almost entirely governed by the highly efficient quadrupolar relaxation active at the site of each ^17^O nucleus. Consequently, the ^17^O signal linewidth could be more reliably interpreted in terms of the structure and dynamics of individual water molecules, rather than the network of water molecules. As shown in Figure 1B, the natural abundance ^17^O signal of H_2_^17^O exhibits various levels of broadening in saturated alkali chloride solutions, with the most pronounced effect observed in LiCl solution where the ^17^O signal linewidth increased from ≈47 Hz in pure water (95 % H_2_O/5 % D_2_O) to 330 Hz. In comparison, the ^17^O signal linewidths in saturated NaCl, KCl, RbCl and CsCl solutions were ≈100, 92, 63 and 61 Hz, respectively. In line with ^1^H NMR data, the 1D natural abundance ^17^O NMR data indicate the cation type‐dependent effects of saturated alkali chloride solutions on water structure and/or dynamics.

### Direct Probing of the Chloride Anion and Corresponding Cations through Quadrupolar NMR

In addition to ^1^H and ^17^O nuclei of water molecules, the alkali chloride solutions contain NMR‐active quadrupolar nuclei of ions (^35^Cl for chloride anion and ^7^Li, ^23^Na, ^39^K, ^87^Rb and ^133^Cs for the corresponding cations; see Table 1), which could be utilized to directly monitor the structure and dynamics of each hydrated ion. To this end, the 1D quadrupolar NMR spectra of the saturated salt solutions were measured and compared with those of reference solutions containing ≈0.12–0.18 m of the corresponding salts. All the quadrupolar nuclei studied here have half‐integer spins (*I*=3/2 for ^7^Li, ^23^Na, ^35^Cl, and ^87^Rb, *I*=5/2 for ^17^O, and *I*=7/2 for ^133^Cs) and, consequently, their NMR signals are comprised of one central transition and *I*−1/2 pairs of satellite transitions. In liquid samples in which unrestricted isotropic rotation of molecules averages out the interaction between quadrupole moments of these nuclei and the local electric field gradients (EFG), the central and satellite transitions become degenerate and therefore a single NMR signal is observed.^[13]^ At small quadrupolar constant (*C*_Q_) values where second‐order quadrupolar interactions are negligibly small, the transverse relaxation of this signal is composed by *I*+1/2 exponential components.^[13a]^ Consequently, the Fourier‐transformed NMR signal will have an *I*+1/2‐Lorentzian (double‐Lorentzian for ^7^Li, ^23^Na, ^35^Cl, and ^87^Rb, triple‐Lorentzian for ^17^O, and quadruple‐Lorentzian for ^133^Cs) shape. However, in the fast extreme‐narrowing regime typically relevant in our experiments, the relaxation rates of corresponding components become identical and consequently a single‐Lorentzian NMR signal is recovered (more details in the Experimental Section).^[13c]^

**Figure 2:**
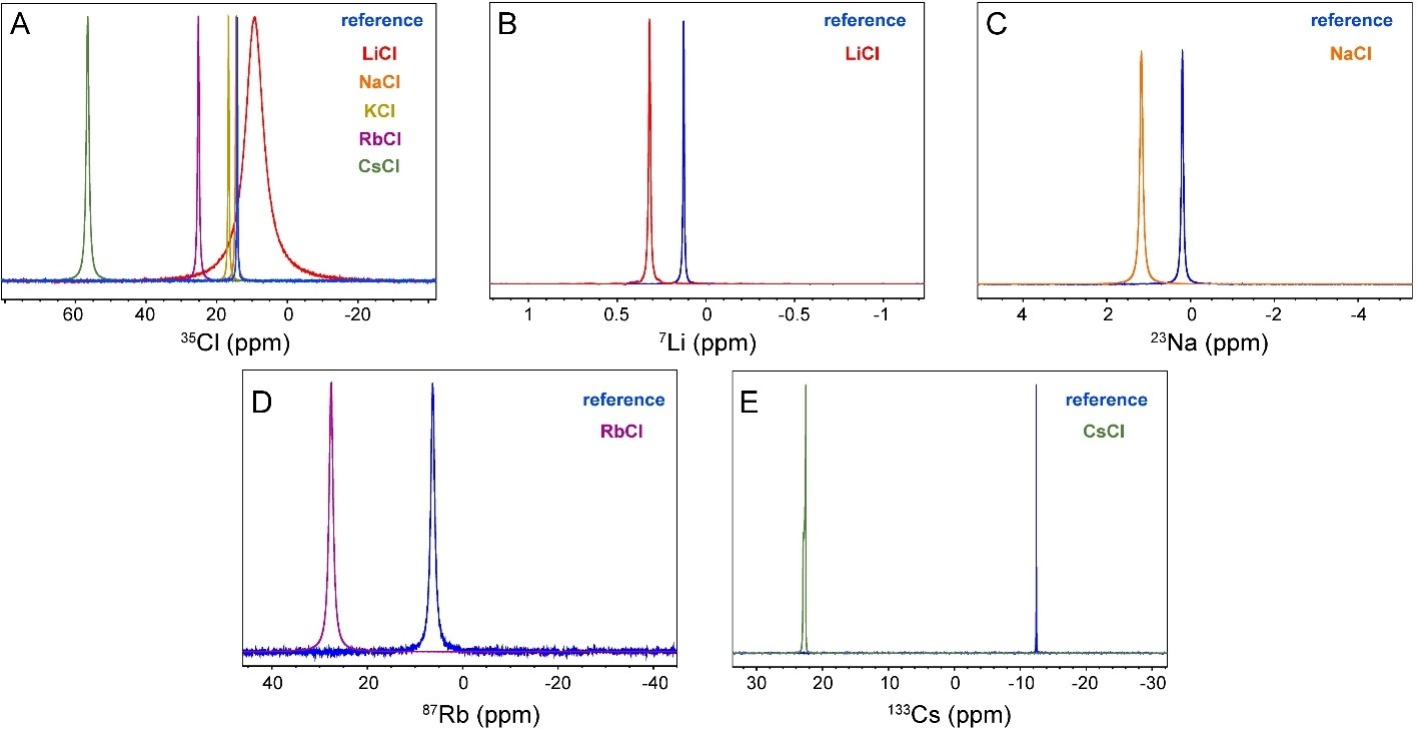
1D spectra of quadrupolar nuclei ^35^Cl (A), ^7^Li (B), ^23^Na (C), ^87^Rb (D) and ^133^Cs (E) in reference, i. e., dilute, and saturated alkali chloride solutions.

Figure 2A shows 1D ^35^Cl spectra of saturated solutions of various alkali chlorides. The linewidth of ^35^Cl signals increased from ≈9 Hz in reference solutions to ≈236, 16, 12, 20 and 35 Hz, respectively, in saturated LiCl, NaCl, KCl, RbCl and CsCl solutions. The change in ^35^Cl linewidth was particularly pronounced in LiCl, while the smallest change was observed for KCl. The larger linewidth of ^35^Cl signals in saturated salt solutions reflect significant alterations in the structure‐dependent EFG and/or dynamics of hydrated chloride anions.

Figures 2B–E respectively show the 1D ^7^Li, ^23^Na, ^87^Rb and ^133^Cs spectra of reference and saturated alkali chloride solutions. No NMR measurement could be performed for the low‐γ ^39^K nuclei in potassium chloride solution, as the ^39^K Larmor frequency was well below the lower frequency limit of the TBO probe of our NMR spectrometer. The signal linewidth of ^7^Li^+^, ^23^Na^+^ and ^87^Rb^+^ ions rose from ≈0.16, 5.7 and 129 Hz, respectively, in reference solutions to 0.88, 9.1 and 131 Hz in corresponding saturated solutions. The spectrum of ^133^Cs^+^ ion in the saturated CsCl solution resembled a powder‐like chemical shift anisotropy (CSA) pattern. However, after some time, the signal was narrowed down to a linewidth of 0.13 Hz identical to observations in its reference solution. This transient behavior was not observed for any other saturated alkali chloride solution studied here and is most likely caused by gradual dissolution of microscopic salt particles. Overall, the NMR signals of cations were influenced by high salt concentration and exhibited varying degrees of broadening in saturated solutions, with largest (relative) broadening observed for ^7^Li^+^ (ca. 5.5‐fold) and ^23^Na^+^ (ca. 1.6‐fold) signals.

### Diffusion of Water within Saturated Salt Solutions

**Figure 3:**
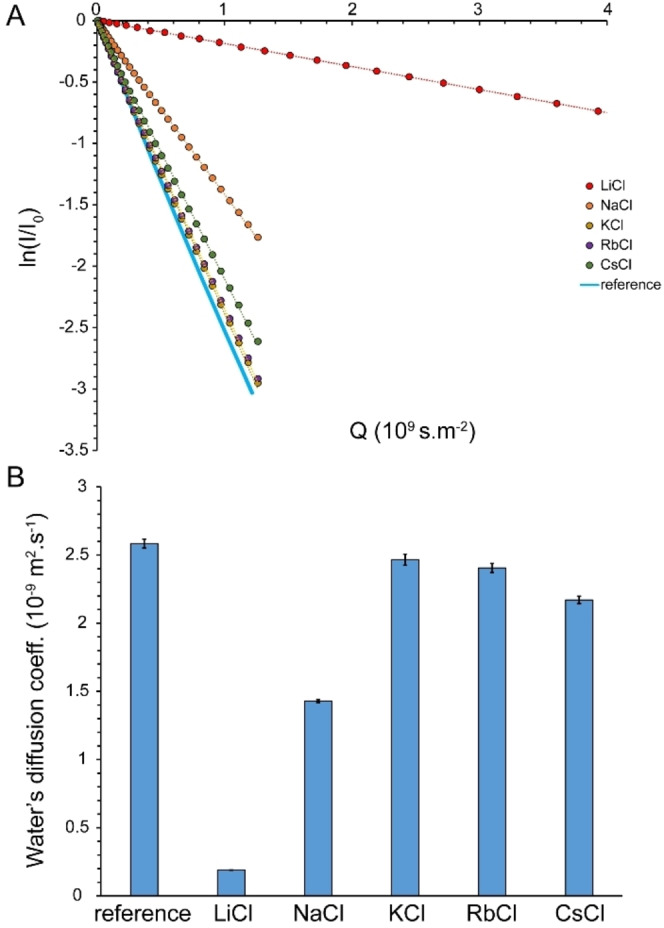
Diffusion coefficient (average±SD) of water in reference sample (95 % H_2_O/5 % D_2_O) and saturated LiCl, NaCl, KCl, RbCl and CsCl solutions, obtained through ^1^H‐based PFG NMR measurements (A, B). The strongest drop in diffusion of water was observed in LiCl followed by NaCl solutions (B).

Diffusion of water molecules within tetrahedrally hydrogen‐bonded structures is largely governed by hydrogen‐bond exchange dynamics, that is, the time constant of breakage and formation of hydrogen bonds, whether it is the H−O⋅⋅⋅H bond in bulk water or O−H⋅⋅⋅X^−^ in the first hydration shell of anions. As stated above, nearly all water molecules in the studied salt solutions are located within the first hydration layer of ions and almost no free water molecules exist between them. To investigate the effect of this water‐ion arrangement on the translational mobility of water molecules, we measured the diffusion coefficient of water through ^1^H PFG NMR diffusion experiments (Figure 3A). In these experiments, the translational mobility of molecules is monitored through application of z‐axis magnetic field gradients, encoding the position of molecules along the z‐axis through modulating their NMR signal frequencies.^[14]^ After proper gradient calibration, the diffusion coefficient, *D*, of pure water (95 % H_2_O/5 % D_2_O) was determined as 2.58±0.03×10^−9^ m^2^ ⋅ s^−1^. Considering the average oxygen–oxygen distance (*l*) of ≈0.28 nm in the radial distribution function of water, the characteristic residence time, τ, of a water molecule at its position within the hydrogen‐bonded structure of water is τ=*l* ^2^/6*D* of about 5 ps. This value is around one order of magnitude longer than the time constant of hydrogen‐bond length fluctuations in pure water, as determined from femtosecond transient vibrational spectroscopy,^[15]^ and in line with some recent reports,^[16]^ indicating that translational jumps of water molecules require slower hydrogen bond exchange dynamics involving hydrogen bond breakage and formation, not mere hydrogen bond fluctuations. In the saturated LiCl solution, the diffusion coefficient of water exhibited a dramatic drop to 0.19±0.00×10^−9^ m^2^ ⋅ s^−1^, reflecting a severe restriction in translational mobility of the water molecules. Considering the ≈20 m concentration of the saturated LiCl solution (Table 2) and a coordination number of 5 for Cl^−^ ions,[^6^, ^7^] it is estimated that ≈20×5=100 m of the 110 m O−H groups of water form O−H⋅⋅⋅Cl^−^ hydrogen bonds and only the remaining 10 m of them form O−H⋅⋅⋅O hydrogen bonds. The average Li^+^−Cl^−^ distance in this solution is 0.22 nm (Table 2), and the residence time of water molecule in the hydration shell of a Cl^−^ ion, defined as the time it takes for a water molecule to diffuse across this distance, is ≈42 ps. A less pronounced decrease was observed in the saturated NaCl solution, in which the diffusion coefficient of water was 1.43±0.01×10^−9^ m^2^ ⋅ s^−1^ and the corresponding residence time was determined to ≈12 ps. The saturated KCl, RbCl and CsCl solutions showed only slight decreases in the diffusion coefficient of water (2.47±0.04×10^−9^, 2.41±0.03×10^−9^ and 2.17±0.03×10^−9^ m^2^ ⋅ s^−1^, respectively) and the correspondingly defined residence times of water molecules were 8, 6 and 5 ps. In line with the ^35^Cl signal linewidths (see above), the smallest change in diffusion coefficient of water was observed for the KCl solution (Figure 3B).

### Diffusion of Ions within Saturated Salt Solutions

**Figure 4:**
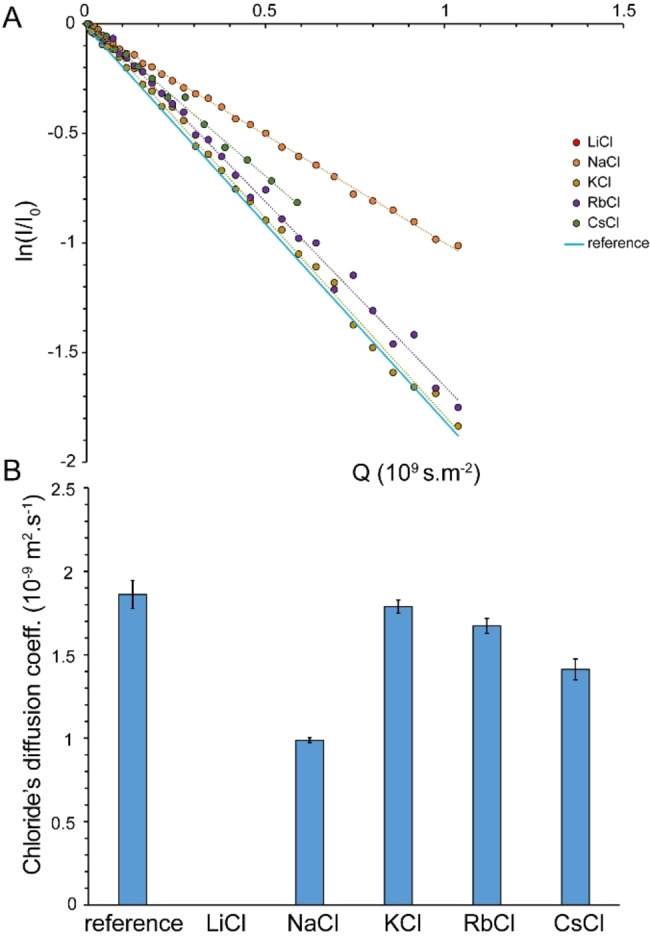
Diffusion coefficient (average±SD) of chloride anion (Cl^−^) in reference and saturated NaCl, KCl, RbCl and CsCl solutions, obtained through ^35^Cl‐based PFG NMR measurements (A, B). The most significant drop in diffusion coefficient of Cl^−^ ion was observed in saturated NaCl solution. Severe ^35^Cl relaxation losses precluded diffusion measurement in saturated LiCl solution (B). Dilute alkali chloride solutions were used as reference.

**Figure 5:**
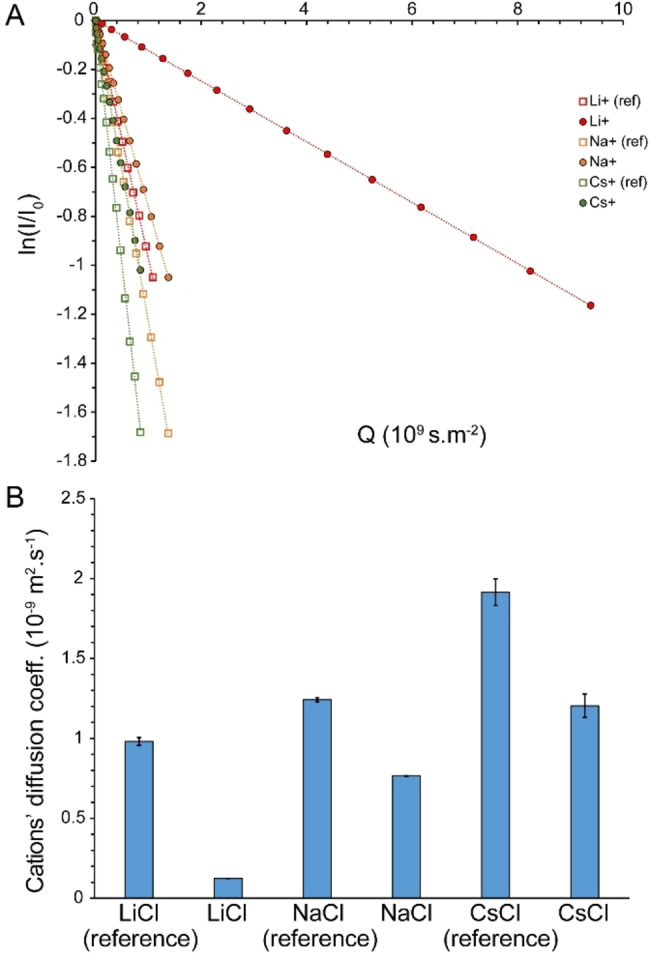
Diffusion coefficient (average±SD) of cations Li^+^, Na^+^ and Cs^+^ in reference and saturated LiCl, NaCl and CsCl solutions, obtained respectively through ^7^Li‐, ^23^Na‐ and ^133^Cs‐based PFG NMR measurements (A, B). The Li^+^ ion exhibited the most significant decrease in translational diffusion (B). Severe Relaxation losses did not allow diffusion measurement for Rb^+^ ion in reference or saturated solutions. Dilute alkali chloride solutions were used as reference.

In saturated alkali chloride solutions, water molecules are mostly bound to chloride anions through O−H⋅⋅⋅Cl^−^ hydrogen bonds and to cations through electrostatic interactions. To examine whether the translational mobility of water molecules and ions becomes correlated under this condition, we subsequently measured the diffusion coefficients of the chloride anion and the corresponding cations through heteronuclear PFG NMR experiments (Figures 4 and 5). The diffusion coefficient of Cl^−^ in the reference alkali chloride solutions was 1.86±0.11×10^−9^ m^2^ ⋅ s^−1^, around 74 % of the diffusion coefficient of water in the same solutions. Severe relaxation losses during diffusion delay (big delta, Δ) precluded Cl^−^ ion diffusion measurement in saturated LiCl solutions. In contrast, the diffusion coefficients of Cl^−^ ions in saturated NaCl, KCl, RbCl and CsCl solutions were measured to 0.99±0.02×10^−9^, 1.79±0.04×10^−9^, 1.67±0.05×10^−9^ and 1.47±0.06×10^−9^ m^2^ ⋅ s^−1^, respectively. The diffusion coefficients of Cl^−^ ions in the saturated salt solutions were clearly distinct from those of the water molecules (see above), indicating that water and chloride ions preserved a significant level of independent translational mobility. Again, as for the ^35^Cl linewidth and water diffusion, the smallest change in diffusion coefficient of Cl^−^ was observed in the saturated KCl solution (Figure 4B).

With regards to diffusion of cations, the diffusion coefficients of Li^+^, Na^+^ and Cs^+^ ions in reference solution were determined to 0.98±0.03×10^−9^, 1.24±0.01×10^−9^ and 1.91±0.08×10^−9^ m^2^ ⋅ s^−1^, respectively (Figures 5A and B). The order of the diffusion coefficients reflects the size of the hydrated ions, which increases with the charge density of monovalent cations and is therefore the biggest for hydrated Li^+^ ions. In saturated solutions, the diffusion coefficients of cations decreased respectively to 0.12±0.03×10^−9^ for Li^+^, 0.77±0.01×10^−9^ for Na^+^ and 1.19±0.07×10^−9^ m^2^ s^−1^ for Cs^+^ (Figures 5A and B).

Due to severe relaxation losses during diffusion delay, no diffusion coefficient could be measured for the Rb^+^ ion in saturated (nor in reference) solutions. Here it is important to note that the cations had diffusion coefficients distinct from water and from the chloride anion in saturated salt solutions, with diffusion coefficients generally following the order water>chloride anion>cation. Compared to the reference solutions, the strongest diminution of ion diffusion was observed in LiCl, followed by NaCl, CsCl, RbCl and KCl solutions (Figures 4B and 5B). For each salt type, however, the diffusion diminution level was rather similar, but not identical, for water, chloride anion and cations. Overall, the diffusion data indicate that except for LiCl and, to a lower extent, NaCl solutions, water molecules retain a large level of translational mobility in saturated alkali chloride solutions. In all these solutions, water mobility remains fairly independent from the chloride anion and corresponding cations.

### Reorientational Mobility of Water Molecules within Saturated Salt Solutions

**Figure 6:**
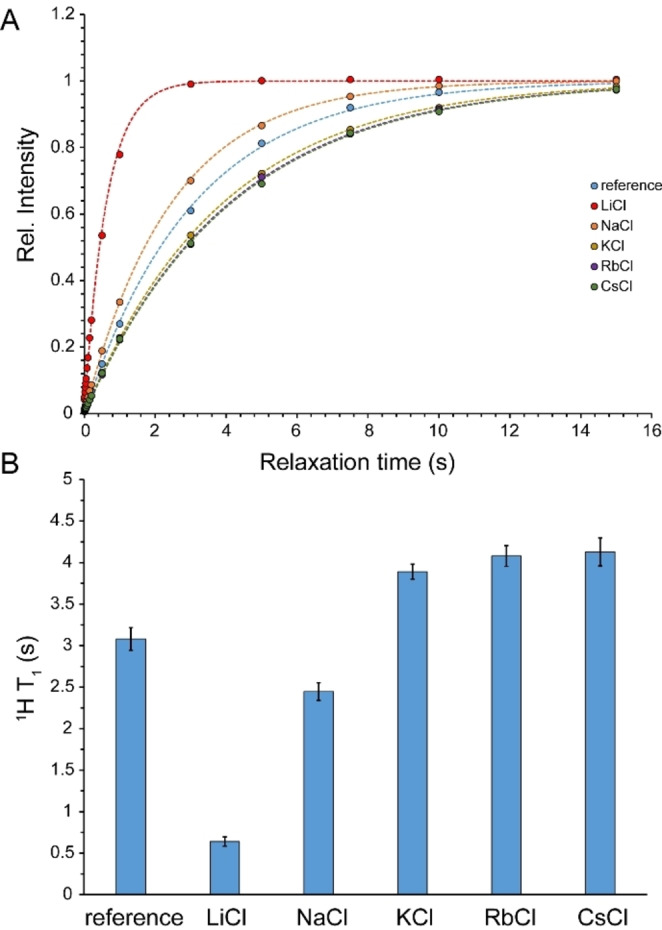
^1^H *T*_1_ relaxation time (average±SD) of water in reference sample (95 % H_2_O/5 % D_2_O) and saturated LiCl, NaCl, KCl, RbCl and CsCl solutions (A, B). The LiCl and NaCl solutions exhibit a shortening of *T*_1_, while KCl, RbCl and CsCl solutions show a prolonged *T*_1_.

Subsequently, we investigated the reorientational mobility of water molecules inside saturated alkali chloride solutions through ^1^H and ^17^O spin‐lattice (*T*_1_) relaxation measurements. As stated above, the *T*_1_ relaxation of water ^1^H in highly concentrated salt solutions is largely governed by intra‐ and intermolecular proton‐proton dipolar coupling and the dipolar coupling between water protons and the NMR‐active nuclei of nearby ions. Due to fluctuations in the distance‐dependent dipolar coupling Hamiltonian of the spin system caused by translational motions, the *T*_1_ relaxation of water protons is sensitive to both reorientational and translational motions of the local network of water molecules and associated ions. It is, however, notable that the dipolar coupling relaxation of ^1^H caused by nearby spin‐*I* nucleus scales with γ_H_^2^γ_I_^2^ *I*(*I*+1)/<*r*_H‐I_>^6^, where γ_I_ is the gyromagnetic ratio of nearby nucleus and <*r*_H‐I_> is the average inter‐nucleus distance. Consequently, the dominant ^1^H relaxation mechanism is the dipolar coupling between neighboring protons. The ^1^H *T*_1_ times of water were measured through saturation‐recovery experiments to avoid radiation damping contributions, which otherwise would significantly reduce ^1^H *T*_1_ of water in inversion‐recovery experiments (Figure 6A). The ^1^H *T*_1_ of pure water (95 % H_2_O/5 % D_2_O) was 3.1±0.1 s, which in saturated LiCl and NaCl solutions decreased to 0.64±0.06 and 2.4±0.1 s, respectively. Interestingly, the ^1^H *T*_1_ of water in saturated KCl, RbCl and CsCl solutions changed in the opposite direction and increased, respectively, to 3.9±0.1, 4.1±0.1 and 4.1±0.2 s (Figure 6B). The larger ^1^H *T*_1_ of water in saturated KCl, RbCl and CsCl solutions are most likely caused by an increase in intermolecular ^1^H−^1^H distances in these solutions, while in NaCl and particularly in LiCl solutions, such changes in intermolecular distances are over‐compensated by counteracting effects of alterations in water reorientational dynamics.

**Figure 7:**
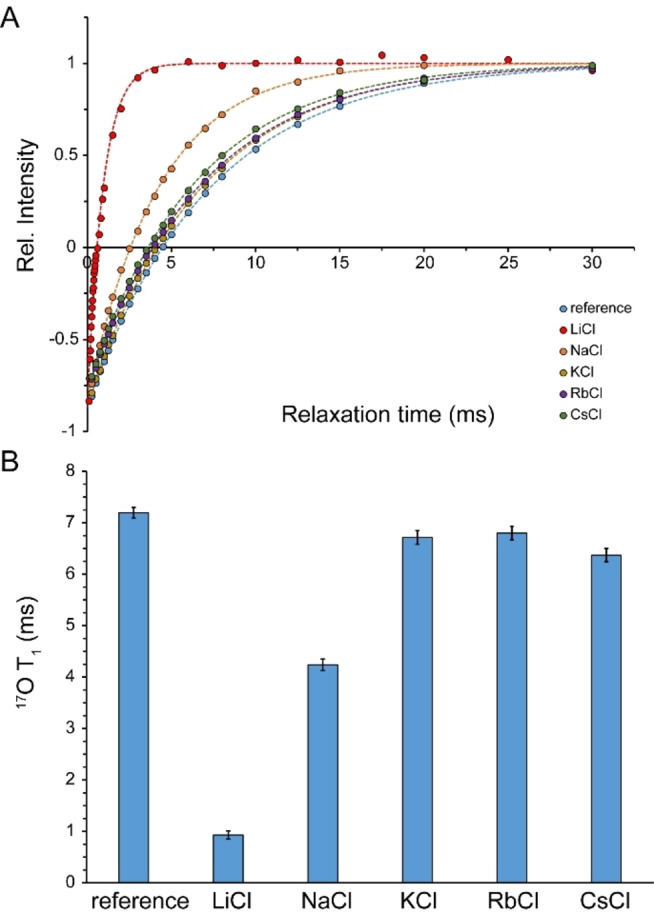
^17^O *T*_1_ relaxation time (average±SD) of water in reference sample (95 % H_2_O/5 % D_2_O) and saturated LiCl, NaCl, KCl, RbCl and CsCl solutions, measured through inversion‐recovery experiments (A, B). The most significant decrease in ^17^O *T*_1_ was observed in LiCl followed by NaCl solutions (B).

The spin‐5/2 ^17^O NMR signal is composed by three components associated with one central transition (|-1/2>↔|+1/2> ) and two pairs of satellite transitions (|±1/2>↔|±3/2> and|±3/2>↔|±5/2> ). In solution and within the fast “extreme narrowing” regime when $\omega_{17O}\tau_{c}<<1$ (τ_c_: rotational correlation time corresponding to the main axis of the EFG tensor at the site of ^17^O nucleus, ω_17O_: Larmor frequency of ^17^O), the three degenerate transitions share the same relaxation rate and consequently a mono‐exponential *T*_1_ relaxation process is observed. The ^17^O *T*_1_ of water were measured through inversion‐recovery experiments, in which mono‐exponential recovery curves typical of extreme narrowing regime were observed (Figure 7A). The ^17^O *T*_1_ of pure water (95 % H_2_O/5 % D_2_O) was 7.2±0.1 ms, in close agreement with previous reports.^[17]^ In saturated LiCl and NaCl solutions, the ^17^O *T*_1_ of water decreased to 0.93±0.08 and 4.2±0.1 ms, respectively, while the corresponding values in saturated KCl, RbCl and CsCl solutions were determined to 6.7±0.1, 6.8±0.1 and 6.4±0.2 ms (Figure 7B).

The *T*_1_ relaxation of quadrupolar ^17^O nuclei is largely governed by the quadrupolar relaxation mechanism, which is sensitive to the EFG present at the site of ^17^O nuclei as well as their reorientational dynamics. While our current data do not allow quantitative disentangling of the effect of structure‐dependent EFG term from dynamical effects, it seems safe to state that the reorientational dynamics of the water molecules are prominently slowed down in saturated LiCl and to lower extent, NaCl solutions. On the other hand, the effects in saturated KCl, RbCl and CsCl solutions appear to be rather small. The restricted reorientational dynamics of water in highly concentrated salt solutions are likely caused by slowing down of the out‐of‐shell rotation of the O−H⋅⋅⋅Cl^−^ system, as the high concentration of ions and its resultant steric hindrance makes the approach of another water molecule and formation of a bifurcated hydrogen‐bonded transition state difficult.

### Reorientational Mobility of Ions within Saturated Salt Solutions

Next, we examined the reorientational dynamics of chloride anions and corresponding cations in saturated alkali chloride solutions through *T*_1_ measurement of quadrupolar ^35^Cl, ^7^Li, ^23^Na, ^87^Rb and ^133^Cs. In addition to the quadrupolar relaxation mechanism, the dipolar coupling between these nuclei and their neighboring nuclei may contribute to their spin‐lattice relaxation, especially in saturated salt solutions where the average distance between high‐spin nuclei is minimized. However, except for nuclei with low quadrupole moments located in nearly symmetric environments such as ^7^Li in LiCl and particularly ^133^Cs in CsCl solutions, the dominant relaxation mechanism is quadrupolar relaxation. Under fast “extreme‐narrowing” regime, the two‐exponential quadrupolar relaxation of spin‐3/2 ^35^Cl, ^7^Li, ^23^Na, and four‐exponential relaxation of spin‐7/2 ^133^Cs signals are expected to be reduced to a mono‐exponential relaxation process, as the rates associated to the single central transition and one (for ^35^Cl, ^7^Li, ^23^Na) or three (for ^133^Cs) pairs of satellite transitions become identical (see Experimental Section for further details).

**Figure 8:**
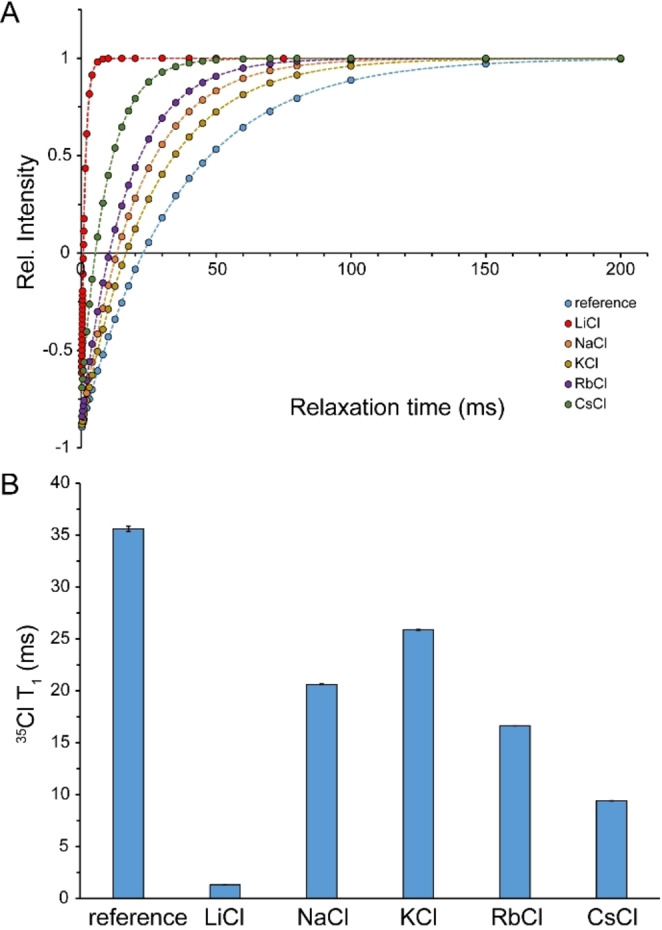
^35^Cl *T*_1_ relaxation time (average±SD) in reference and saturated LiCl, NaCl, KCl, RbCl and CsCl solutions, measured through inversion‐recovery experiments (A, B). Dilute alkali chloride solutions were used as reference.

The *T*_1_ of ^35^Cl in reference solutions was 35.4±0.3 ms, which in saturated LiCl, NaCl, KCl, RbCl and CsCl solutions decreased respectively to 1.33±0.01, 20.63±0.02, 25.88±0.03, 16.64±0.01 and 9.41±0.01 ms (Figures 8A and B). The shorter *T*_1_ of ^35^Cl at higher concentrations is consistent with the expected slowing down of the out‐of‐shell rotation of O−H⋅⋅⋅Cl^−^ hydrogen bonds. The shortest ^35^Cl *T*_1_ was observed in saturated LiCl solution. Surprisingly however, the next smallest ^35^Cl *T*_1_ were observed not in NaCl, but in CsCl and RbCl, respectively. The unexpected trend of ^35^Cl *T*_1_ values are likely to reflect changes in the structure‐ dependent EFG tensors existing at the site of ^35^Cl nuclei, rather than their reorientational dynamics.

**Figure 9:**
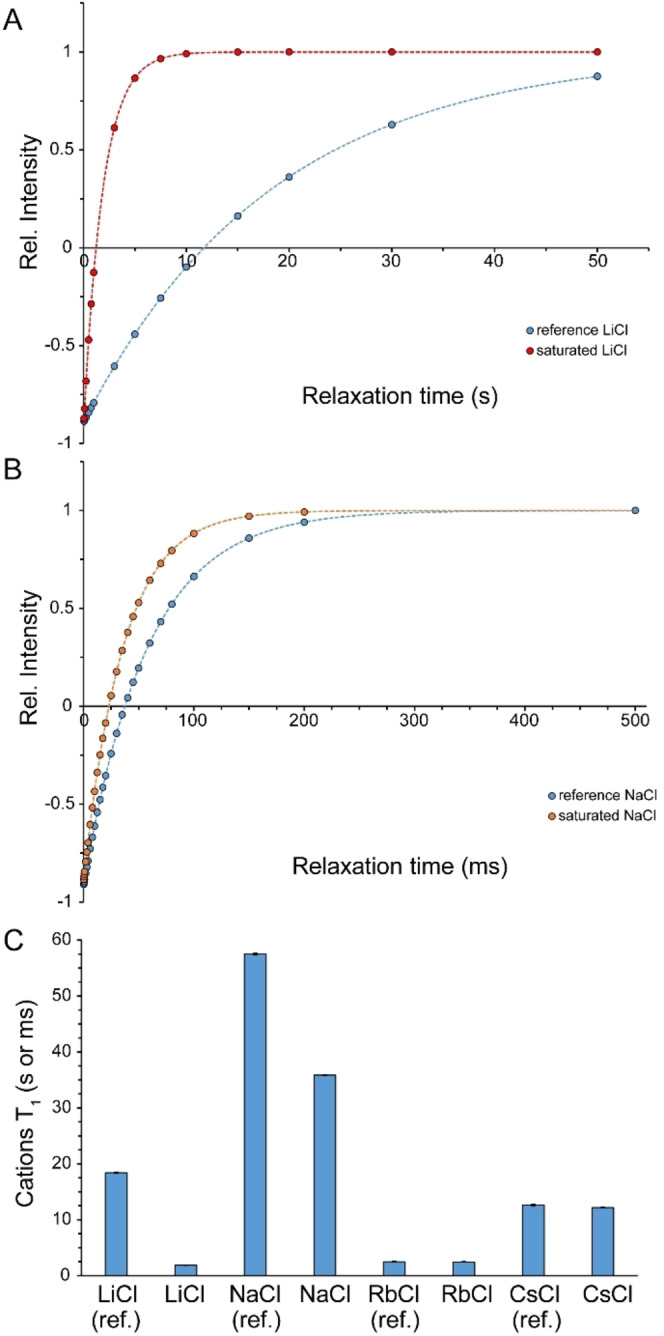
*T*_1_ relaxation time (average±SD) of cations in reference and saturated LiCl, NaCl, RbCl and CsCl solutions, measured through inversion‐recovery experiments (A, B, C). In (C), *T*_1_ of ^7^Li^+^ and ^133^Cs^+^ are shown in seconds, while those of ^23^Na and ^87^Rb are given in ms. The most significant *T*_1_ decreases were observed in LiCl followed by NaCl solutions. Dilute alkali chloride solutions were used as reference. See also Figure S1 (Supporting Information).

With regards to cations, the *T*_1_ of ^7^Li, ^23^Na, ^87^Rb and ^133^Cs changed respectively from 18.4±0.1 s, 57.5±0.1 ms, 2.50±0.01 ms and 12.6±0.1 s in reference solutions to 1.9±0.1 s, 35.9±0.1 ms, 2.49±0.01 ms and 12.2±0.2 s in saturated LiCl, NaCl, RbCl and CsCl solutions. The most pronounced decreases were observed for Li^+^ and then Na^+^ ions, while Rb^+^ and Cs^+^ ions did not show any significant change (Figures 9 and S1, Supporting Information). These observations are consistent with a significant level of reorientational mobility restriction for Li^+^ and Na^+^ ions, while Rb^+^ and Cs^+^ ions seem to have preserved their reorientational mobility in saturated solutions.

### Activation Energies of Water Molecules and Ions within Saturated NaCl Solution

**Figure 10:**
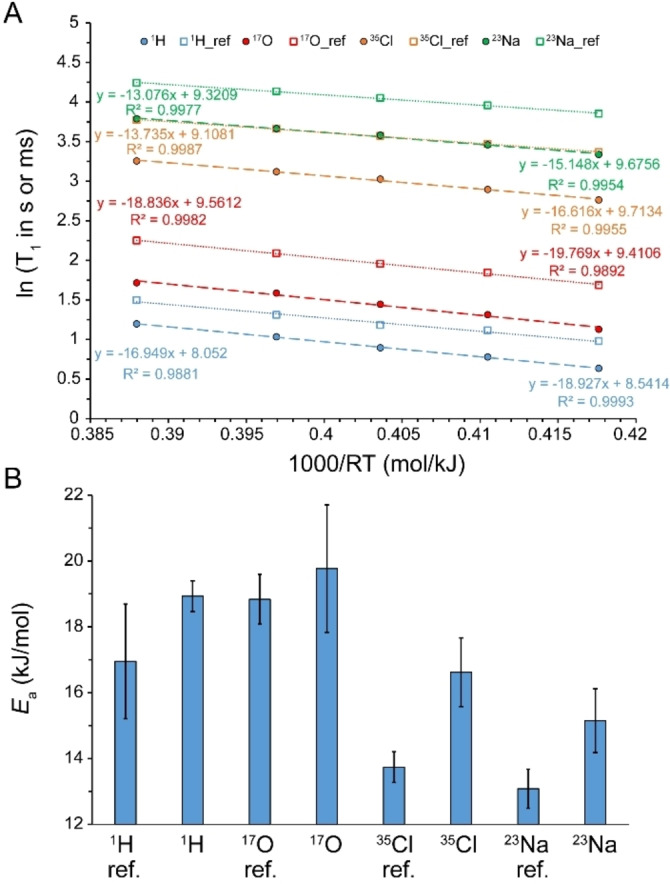
(A) Arrhenius analysis of temperature dependence of *T*_1_ relaxation time for water molecules (^1^H and ^17^O) and chloride (^35^Cl) and sodium (^23^Na) ions in reference (dilute) and saturated NaCl solutions. (B) Comparison of *T*_1_‐based activation energies (*E*_a_; average±SD) obtained for reorientational dynamics of water molecules (^1^H and ^17^O) and chloride (^35^Cl) and sodium (^23^Na) ions. Except for ^1^H, all other *T*_1_ values in (A) are given in ms.

To further examine the possibility of concerted motions in saturated salt solutions, we studied the temperature dependence of waters’ and ions’ translational and rotational motions and, through Arrhenius analysis, derived the corresponding activation energies. As shown recently, the activation energies could be used to gain mechanistic insight on water dynamics and distinguish among its various modes of motion.^[16]^ To this end, we chose NaCl because the water solubility of NaCl shows only weak temperature dependence.^[18]^ Consequently, it is safe to assume that the structure of water and hydrated ions, hence the EFG tensors present at the site of quadrupolar ^17^O, ^23^Na and ^35^Cl in saturated NaCl solutions are fairly constant in the studied temperature range of 288–310 K and therefore the temperature dependence of ^17^O, ^23^Na and ^35^Cl *T*_1_ could be more reliably attributed to water molecules’ and ions’ reorientational mobility changes. Another reason for this choice was that in saturated NaCl solution all the measured probes of (translational and rotational) mobility of water molecule and ions were 57±4 % of their reference values. This observation may be taken to suggest that the motions of water molecules and chloride and sodium ions are coupled to each other through a common mechanism. In reference NaCl solution, the activation energy (*E*_a_) of translational motion of water molecules and sodium and chloride ions were respectively 18180±2638, 18580±870 and 18070±607 J ⋅ mol^−1^ (Figure S2A, Supporting Information). The corresponding values in saturated NaCl solution were 19040±980, 19500±872 and 18190±472 J ⋅ mol^−1^, which within the experimental range of error were not different from reference values and from each other (Supporting Figure S2B). On the other hand, the *E*_a_ of reorientational motion of water molecules and sodium and chloride ions in reference NaCl solution were 16950±1740, 18840±758, 13080±592 and 13740±467 J ⋅ mol^−1^, respectively, based on ^1^H, ^17^O, ^23^Na and ^35^Cl *T*_1_ (Figure 10A). The corresponding values in saturated NaCl solution were 18930±469 for ^1^H, 19770±1939 for ^17^O, 15150±969 for ^23^Na and 16620±1043 J ⋅ mol^−1^ for ^35^Cl, which for ^1^H were in good agreement with previous reports.^[19]^ The *E*_a_ values in saturated NaCl solution are considerably larger than the reference values, reflecting the relatively restricted motion of water molecules and ions inside the saturated solution. Importantly, the *E*_a_ values associated to reorientational dynamics of water molecules and chloride and sodium ions remained distinct from each other even in saturated NaCl solution (Figure 10B), indicating that water molecules and chloride and sodium ions retain a large level of their independent mobility in spite of the water molecules‐ion arrangement dictated by the high NaCl concentration.

## Discussion

^1^H‐based NMR diffusion measurements of water in saturated alkali chloride solutions indicated the important role of cation type in determining translational mobility of water within such confined water molecules‐ions arrangements: water mobility was restricted by a factor of ≈13.6 in the presence of Li^+^ and ≈1.8 in the presence of Na^+^, while only little restriction was observed in the presence of K^+^, Rb^+^ and Cs^+^. This is in qualitative agreement with the known effect of cations on the energy dynamics of anionic hydration shells as revealed in cation dependence of vibrational lifetimes of water in high concentrated salt solutions. As reflected in the characteristic time of water diffusion being around an order of magnitude longer than the vibrational lifetime of water (see above) and the difference in their activation energies,^[16]^ vibrational relaxation and translational diffusion of water seem to be controlled by distinct mechanisms. Nevertheless, their cation‐type dependence can be explained by the same mechanism: the higher electric field exerted by smaller monovalent cations such as Na^+^ and particularly Li^+^ polarize the O−H⋅⋅⋅O and O−H⋅⋅⋅X^−^ hydrogen bonds more strongly. In regards to diffusion, the resultant strengthening of hydrogen bonds decreases hydrogen bond exchange dynamics and reduces water diffusion. A similar mechanism leads to severe restriction in the reorientational dynamics of water molecules and chloride anions inside saturated LiCl solution and underlies the pronounced broadening of ^1^H, ^17^O and ^35^Cl signals (Figures 1A, B and 2 A). It is notable that this mechanism depends on the finite, albeit modest, polarizability of water molecules, and becomes most efficient when the average cation–water and cation–anion distances are the smallest, as in the saturated LiCl solution (Table 2).

Multinuclear NMR diffusion measurements of saturated alkali chloride solutions allowed direct comparison of diffusion coefficient of water molecules, Cl^−^ anions and corresponding cations. The average cation–anion distance in these highly concentrated salt solutions can accommodate only one shared hydration layer, which suggests that the solvent‐shared ion pairs may diffuse as a whole unit. Indeed, such behavior has been recently reported in ternary water‐in‐salt solutions of LiCl−CsCl−D_2_O, where most of the molecules and ions seem to move as dynamic clusters named “cybotactic groups”.^[20]^ Contrary to this expectation, our NMR diffusion data provides distinct diffusion coefficients for water molecules, the Cl^−^ anion and corresponding cations, indicating their independent translational dynamics in saturated salt solutions.

The *T*_1_ relaxation times of nuclei with large quadrupolar moments and/or located in highly asymmetric environments are predominantly governed by quadrupolar relaxation mechanism, which, unlike the dipolar coupling mechanism, does not directly depend on intermolecular distances and therefore is not modulated by translational dynamics of molecules. Consequently, the *T*_1_ relaxation times of these nuclei could, in principle, represent the reorientational dynamics of molecules more accurately than those of dipolar coupling‐dominated nuclei. The dynamical interpretation of quadrupolar *T*_1_ relaxation times however requires disentangling the dynamical contribution from the structure‐dependent EFG term, which is often not possible due to lack of structural knowledge. Nonetheless, the *T*_1_ measurements of ^17^O, ^35^Cl and ^23^Na in saturated NaCl concentration over the temperature range of 288–310 K allowed separating the supposedly temperature‐invariant EFG term from dynamical contributions and enabled calculating activation energies for the reorientational dynamics of water molecules and chloride and sodium ions. The distinct activation energies associated with *T*_1_ of ^17^O, ^35^Cl and ^23^Na support the independent reorientational dynamics of water molecules and chloride and sodium ions within saturated NaCl solutions. In general, the multinuclear *T*_1_ relaxation data presented here are consistent with the picture that water molecules and ions preserve a large level of reorientational dynamics in saturated KCl, RbCl and CsCl solutions, but experience partially restricted dynamics in saturated NaCl and particularly LiCl solutions.

Protein motions are strongly coupled to water dynamics at temperatures above 160 K.^[21]^ Changes in water dynamics, for example as a consequence of the highly crowded and confined cellular environments, can therefore affect proteins dynamics and alter their function. Recent ^17^O‐ and ^23^Na‐based NMR studies, however, demonstrate that water molecules and sodium ions retain a high level of their translational and rotational mobility within phase‐separated biomolecular condensates.[^17b^, ^22^] Similarly, except for LiCl, our data show that water molecules and ions remain largely mobile within the highly constrained environment of saturated alkali chloride solutions. These data suggest a large level of water dynamics in the interior of phase‐separated cellular compartments enriched by a high concentration of charged biomolecules such as RNAs. The proposed hypothesis remains to be tested in future studies.

Another notable finding of this study is that water mobility remains nearly intact in saturated KCl solution. The unique mobility of water in a saturated KCl solution is probably related to its average cation–anion distance, which is the largest among the studied alkali chlorides (Table 2). Accordingly, we speculate that the concentration‐invariance of water mobility in KCl solutions may be connected to the origination of life within highly confined K^+^‐enriched environments on early earth and underlie the strikingly large intracellular K^+^/Na^+^ ratio of extant cells.^[23]^

## Conclusion

Following a multi‐nuclear NMR approach including quadrupolar nuclei, we have monitored translational and reorientational mobility of water molecules and ions in saturated solutions of a series of alkali chloride salts. While water molecules‐ions arrangement implies no free interstitial water outside the shared hydration shells of ions, our data clearly show independent translational diffusion of water molecules and ions in these solutions. In addition, temperature‐dependent NMR relaxation data allowed determining activation energies for reorientational motion of water molecules and sodium and chloride ions in saturated sodium chloride solution and supported their independent reorientational dynamics. Except for lithium chloride and to lower extent sodium chloride solutions, our data demonstrated that water molecules retain a large level of mobility inside saturated alkali chloride solutions, in particular in saturated potassium chloride solution, in which water mobility remains nearly intact when compared with pure water or dilute salt solution.

## Experimental Section

### NMR Experiments

NMR experiments were performed at a Bruker spectrometer with proton Larmor frequency of 400.13 MHz. The spectrometer was equipped with a room‐temperature triple resonance broadband (TBO) probe, where for 7Li‐, ^17^O‐, ^23^Na‐, ^35^Cl‐, ^87^Rb‐ and ^133^Cs‐detected experiments the inner coil of the probe was tuned and matched at their corresponding Larmor frequencies. The ^1^H‐detected experiments were measured using the outer coil of the TBO probe. The NMR samples contained saturated salt solutions (see Table 1) in 95 %/5 % (v/v) H_2_O/D_2_O, and the deuteron signal of HDO was used both for frequency locking and the chemical shift reference (4.700 ppm). As reference salt samples, samples with circa 120–180 mm salt concentrations were used. The temperature was set at 298.0 K, unless specified otherwise, and controlled to ±0.05 K using the Bruker VT unit calibrated using the residual proton signals of a standard deuterated methanol sample.

Diffusion coefficients (*D*) of water molecules and Cl^−^, Li^+^, Na^+^ and Cs^+^ ions were determined through ^1^H‐, ^35^Cl‐, ^7^Li‐, ^23^Na‐ and ^133^Cs‐based pulsed‐field‐gradient (PFG) NMR experiments using standard stimulation‐echo based pulse sequence stebpgp1 s. Gradient calibration was performed using the known diffusion coefficient of residual HDO in 99.8 % D_2_O at 298.14 K (1.900×10^−9^ m^2^ ⋅ s^−1^). The gradient‐based intensity attenuation data were fitted to standard Stejskal–Tanner (ST) equation [Eq. 1],

(1) $$I=I_{0}\exp\left(-DQ\right)$$

with $Q=\gamma^{2}\delta^{2}\left(\Delta -\delta /3\right)g^{2}$ , where γ is gyromagnetic ratio of corresponding nucleus, *g* is gradient strength and little and big delta (δ, Δ) are diffusion delays. The details of experimental parameters are provided in supporting Table S1.

The *T*_1_ of ^7^Li, ^17^O, ^23^Na, ^35^Cl, ^87^Rb and ^133^Cs were measured using standard inversion‐recovery experiments. The inversion‐recovery data were well fit to a single‐exponential equation (Eq. 2,

(2) $$I=a(1-2be^{-t/T_{1}})$$

suggesting that reorientational motions of quadrupolar ions occurred within fast “extreme narrowing” regime (see below for more details about relaxation of quadrupolar half‐integer nuclei within fast, slow and ultra‐slow regimes and Table S2, Supporting Information, for experimental details). Further support for the single‐exponential relaxation behaviour of quadrupolar nuclei was provided by lack of signals in longitudinal multi‐quantum‐filtered experiments. The ^1^H *T*_1_ was measured through saturation‐recovery experiments in order to avoid radiation damping issue, and the integrated areas, that is, the first point of FIDs, instead of peak heights, were used for fitting.^[11]^ A continuous‐wave (cw) irradiation at 100 Hz RF strength applied during recycle delay (d1) was used for 1H saturation (further details of experimental parameters in Table S3, Supporting Information), and the recovered intensities were fit to the following Equation 3,

(3) $$I=a(1-e^{-t/T_{1}})$$

The activation energies (*E*_a_) associated to translational diffusion and reorientational dynamics of water molecules and different ions were determined through Arrhenius analysis of temperature‐dependent diffusion coefficients and *T*_1_ values, according to Equation 4:

(4) $$\ln\left(D\right)\;or\;\ln\left(T_{1}\right)=\ln\left(A\right)-E_{a}/RT$$

where *T* is the temperature in K, *R* is the gas constant and *A* is the Arrhenius pre‐factor.

### Relaxation of Quadrupolar Half‐Integer Nuclear Spins in Solution

Except for ^1^H which is a spin‐1/2 nucleus with zero electric quadrupole, all the other nuclei studied here are quadrupolar with half‐integer spins of *I*=3/2 (for ^23^Na, ^35^Cl and ^87^Rb), 5/2 (for ^17^O) and 7/2 (for ^133^Cs). In general, the NMR signal of a half‐integer quadrupolar nucleus with spin *I* is comprised of a superposition of *I*+1/2 single‐quantum transitions, one central transition (|-1/2>↔|+1/2> ) and *I*−1/2 pairs of satellite transitions (e. g. for *I*=7/2 ^133^Cs, three pairs of satellite transitions: |±1/2>↔|±3/2> , |±3/2>↔|±5/2> and |±5/2>↔|±7/2> ). The NMR relaxation of these quadrupolar nuclei are governed by the interaction of their quadrupole moment (Q), representing the prolate‐ (positive Q) or oblate‐like (negative Q) charge distribution inside them, with the electric field gradient (EFG) present at the site of nuclei.^[13b]^ Because of the traceless character of the nuclear quadrupolar interaction and its averaging out due to fast unrestrained molecular motions, the central and satellite transitions become degenerate in solutions.[^13b^, ^13c^, ^24^] According to Redfield's relaxation theory, the quadrupolar interaction‐induced relaxation of half‐integer quadrupolar spins contains *I*+1/2 exponential components, one for the central transition and the others for different pairs of satellite transitions.[^13c^, ^25^] However, under fast motion regime (“extreme narrowing” condition, which is relevant in the current study) where $\omega_{0}\tau_{c}\ll 1$ (ω_0_: Larmor's frequency, τ_c_: rotational correlation time), all the components share the same relaxation times, as follows [Eq. 5]:

(5) $$\frac{1}{T_{1}}=\frac{1}{T_{2}}=\frac{3\pi^{2}}{10}\left(\frac{2I+3}{I^{2}\left(2I-1\right)}\right)\chi^{2}\left(1+\frac{\eta^{2}}{3}\right)\tau_{c}$$

where *I* is the spin quantum number, $\chi =\frac{e^{2}Qq_{zz}}{h}$ is quadrupole coupling constant (C_Q_) and η is the asymmetry parameter, describing the deviation of the EFG tensor, *q*, from axial symmetry. It is however notable that the multi‐exponential relaxation of quadrupolar nuclei is recovered in the slow motion regime where $\omega_{0}\tau_{c}\geq 1$ ,[^13c^, ^25^] and their relaxation deviates from Redfield's theory predictions when molecular motion is further slowed down and the systems enters the so‐called ultraslow motion regime where $\omega_{0}\tau_{c}>{\left(\frac{\omega_{0}}{\omega_{Q}}\right)}^{2}$ (ω_Q_ is quadrupolar coupling constant C_Q_ in angular frequency units).[^13c^, ^26^]

### Estimation of Inter‐Ion Distances

The average cation–cation and anion–anion distances (<*r*>, in m) were estimated from salt concentration (*C*_m_, in m) through

(6) $$<r>=\sqrt[{3}]{\frac{1000}{N_{A}C_{M}}}\;$$

where *N*_A_ is Avogadro's number. The average cation–anion distances were considered as half of <*r*>.

## Conflict of interest

The authors declare no conflict of interest.

1

## Supporting information

As a service to our authors and readers, this journal provides supporting information supplied by the authors. Such materials are peer reviewed and may be re‐organized for online delivery, but are not copy‐edited or typeset. Technical support issues arising from supporting information (other than missing files) should be addressed to the authors.

Supporting Information

## Data Availability

The data that support the findings of this study are available from the corresponding author upon reasonable request.
