# Supplementary material for: Water Dynamics in Highly Concentrated Salt Solutions: A Multi‐Nuclear NMR Approach
Source: ChemistryOpen. 2022 May 31;11(6):e202200080. doi: 10.1002/open.202200080 (PMC9156811; doi:10.1002/open.202200080)
Supplement: Supplementary file 1 — Supporting Information [file OPEN-11-e202200080-s001.pdf]

ChemistryOpen

Supporting Information

Water Dynamics in Highly Concentrated Salt Solutions: A Multi-Nuclear NMR Approach

Nasrollah Rezaei-Ghaleh*

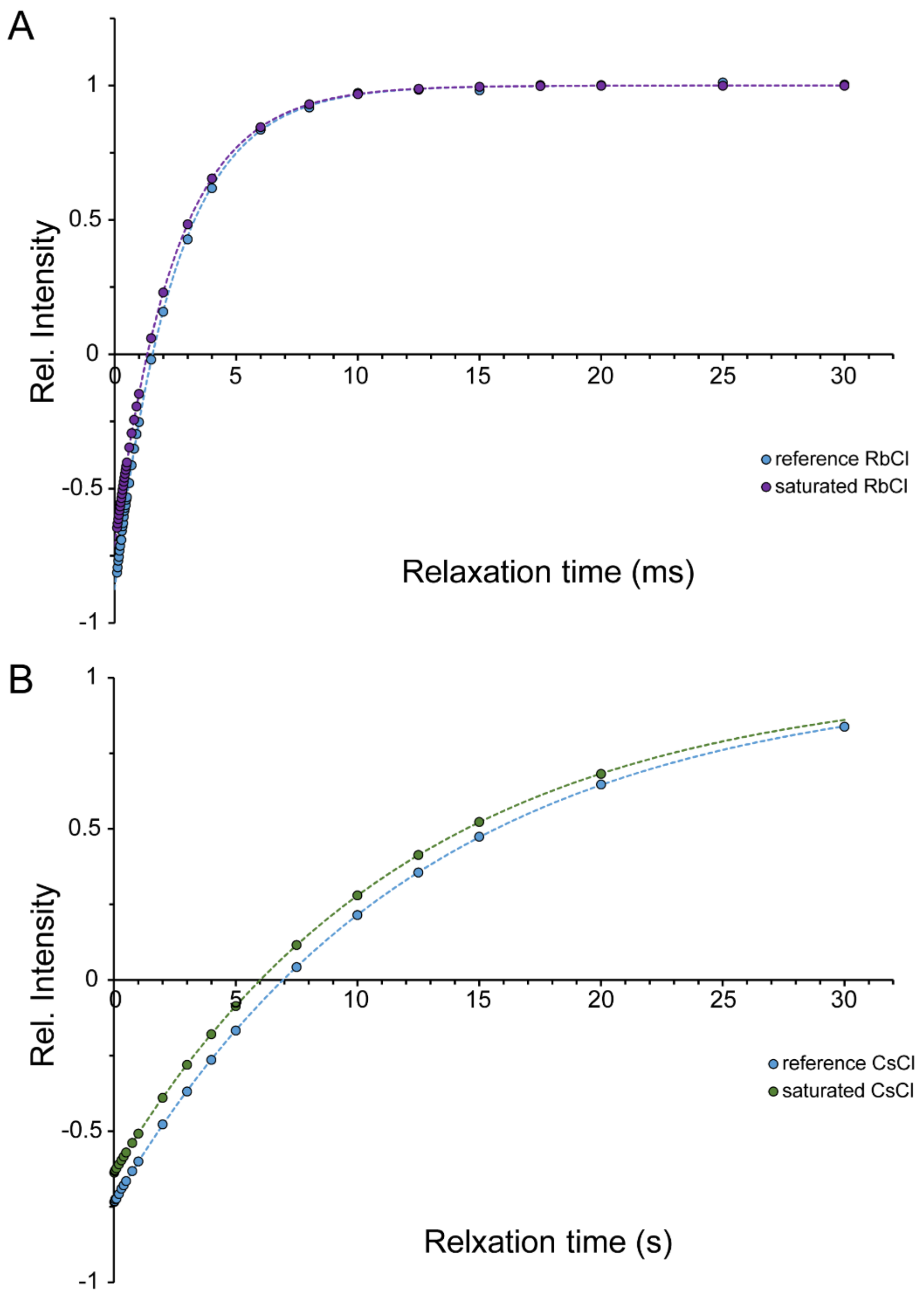

Figure S1. T_1 relaxation time measurements of ^{87}Rb and ^{133}Cs cations in reference, i.e. dilute, and saturated RbCl and CsCl solutions, through inversion-recovery experiments.

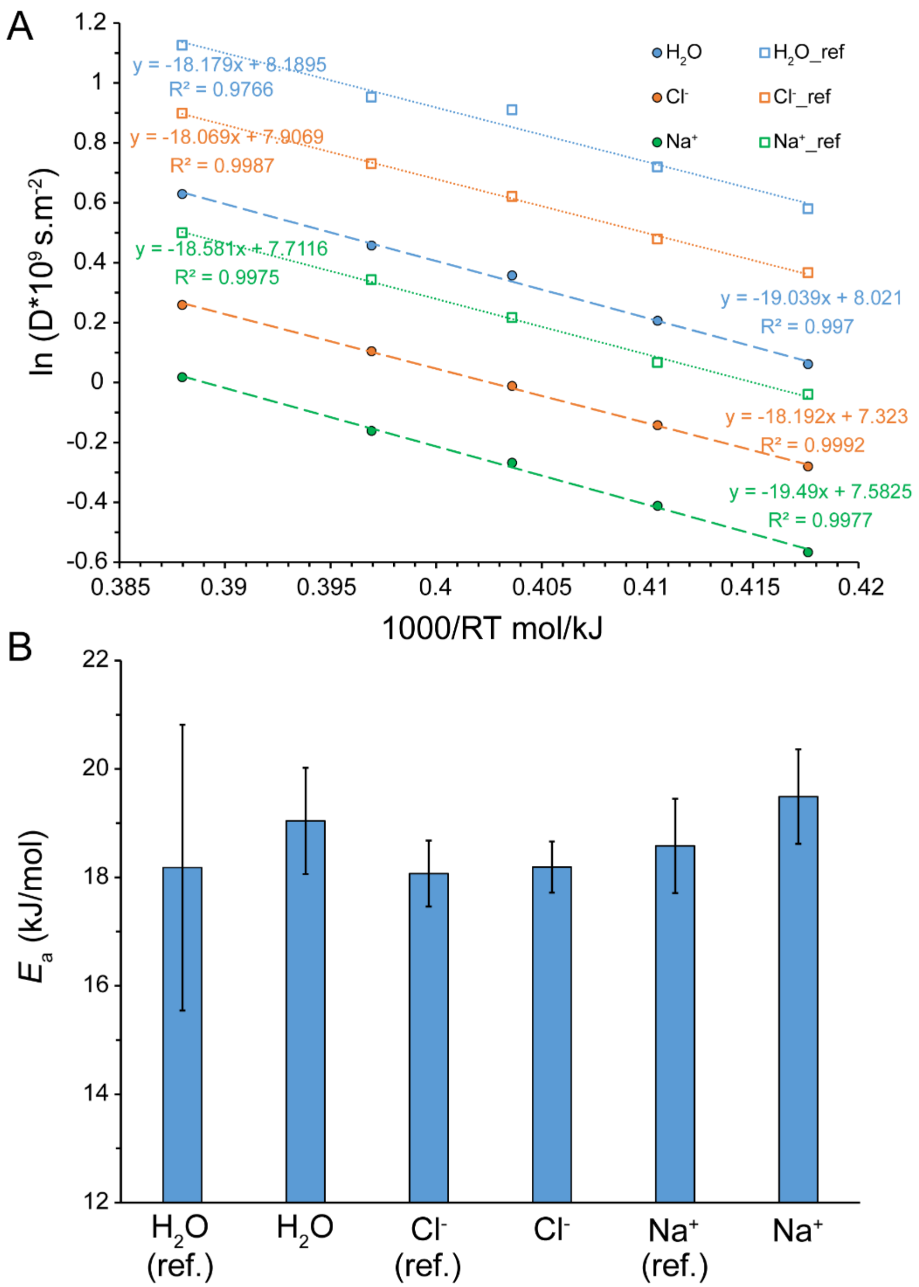

Figure S2. (A) Arrhenius analysis of temperature dependence of diffusion coefficients of water and chloride and sodium ions in reference (dilute) and saturated NaCl solutions. (B) Comparison of activation energies (E_a) obtained for diffusion of water and chloride and sodium ions.

Supporting Table S1. Parameters of Pulsed Field Gradient (PFG)-NMR experiments used for the measurement of diffusion coefficients of water and ions in saturated (reference) alkali chloride solutions.

		Δ in ms*	δ in ms*	No. of gradient points*	No. of NMR scans (NS) *	Recycle delay (d1+acq) in sec*
water	^{1}H	10	2	32	16	3
	-	-	-	-	-	-
	-	-	-	-	-	-
LiCl	^{1}H	20	2	32	16	3
	^{7}Li	50	9 (3)	16	16 (32)	5.6
	^{35}Cl	50	12	32 (16)	128 (1k)	0.7
Nacl	^{1}H	20	2	32	16	3
	^{23}Na	50	5	16	128	1
	^{35}Cl	50	12	32 (16)	512	0.7
KCl	^{1}H	20	2	32	16	3
	^{39}K	-	-	-	-	-
	^{35}Cl	50	12	32 (16)	128 (1k)	0.7
RbCl	^{1}H	20	2	32	16	3
	^{87}Rb	-	-	-	-	-
	^{35}Cl	50	12	32 (16)	512 (1k)	0.7
CsCl	^{1}H	20	2	32	16	3
	^{133}Cs	50	8	16	16 (48)	3.8
	^{35}Cl	20 (50)	16 (12)	16	256 (1k)	0.7

*. If the values used in reference experiments were different from those of main experiments, they are shown in parentheses.

Supporting Table S2. Parameters of inversion-recovery experiments used for the measurement of ^{17}O, ^{7}Li, ^{23}Na, ^{35}Cl, ^{87}Rb and ^{133}Cs T_1 in saturated (reference) alkali chloride solutions.

		Range of relaxation delays in ms	No. of relaxation datapoints *	No. of NMR scans (NS) *	Recycle delay (d1+acq) in sec*
water	^{17}O	0.25-30	21	2560	0.7
	-	-	-	-	-
	-	-	-	-	-
LiCl	^{17}O	0.1-30 (0.25-30)	35 (21)	3072 (2560)	0.7
	^{7}Li	50-50000	14	8	63
	^{35}Cl	0.1-200	38	128	0.5
Nacl	^{17}O	0.25-30	21	2560	0.7
	^{23}Na	0.25-200 (0.25-500)	26 (27)	64	1.3
	^{35}Cl	0.25-200	26	128	0.7
KCl	^{17}O	0.25-30	21	3072 (2560)	0.7
	^{39}K	-	-	-	-
	^{35}Cl	0.25-200	26	64 (256)	0.7
RbCl	^{17}O	0.25-30	21	3072 (2816)	0.7
	^{87}Rb	0.1-30	35	64 (128)	0.6
	^{35}Cl	0.25-200	26	128 (256)	0.7
CsCl	^{17}O	0.25-30	21	3072 (2176)	0.7
	^{133}Cs	10-20000	19	8	21
	^{35}Cl	0.25-200	26	128 (256)	0.7

*. If the values used in reference experiments were different from those of main experiments, they are shown in parentheses.

Supporting Table S3. Parameters of saturation-recovery experiments used for the measurement of ^{1}H T_1 in saturated and reference alkali chloride solutions.

		Range of relaxation delays in sec	No. of relaxation datapoints *	No. of NMR scans (NS) *	d_1 in sec*
water	^{1}H	0.005-15	17	8	30
LiCl	^{1}H	0.005-15	17	8	30
Nacl	^{1}H	0.005-15	17	8	30
KCl	^{1}H	0.005-15	17	8	30
RbCl	^{1}H	0.005-15	17	8	30
CsCl	^{1}H	0.005-15	17	8	30
